# Pacemaker Implantation Associated Myocardial Micro-Damage: A Randomised Comparison between Active and Passive Fixation Leads

**DOI:** 10.1038/s41598-018-23209-5

**Published:** 2018-03-20

**Authors:** Patrick Blažek, Jerko Ferri-Certić, Hrvoje Vražić, Carsten Lennerz, Christian Grebmer, Kazuaki Kaitani, Martin Karch, Boris Starčević, Verena Semmler, Christof Kolb

**Affiliations:** 1 Deutsches Herzzentrum München, Klinik für Herz- und Kreislauferkrankungen, Abteilung für Elektrophysiologie, Fakultät für Medizin der Technischen Universität München, Munich, Germany; 2 Dubrovnik General Hospital, Dept. of Cardiology, Dubrovnik, Croatia; 3 University Hospital Dubrava, Division of Cardiology, Department of Internal Medicine, Zagreb, Croatia; 4 Tenri Hospital, Dept. of Cardiology, Tenri, Japan; 5 Klinikverbund Kempten-Oberallgäu, Abteilung für Kardiologie, Kempten, Germany; 6 Present Address: DZHK (German Centre for Cardiovascular Research), partner site Munich Heart Alliance, Munich, Germany

## Abstract

Fixation of the pacemaker leads during pacemaker implantation leads to an increase of cardiac Troponin T (cTnT) that can be interpreted as a sign of minimal myocardial damage. This trial evaluates whether the mechanism type of lead fixation influences the magnitude of cTnT release. Patients having a *de-novo* cardiac pacemaker implantation or a lead revision were centrally randomized to receive either a ventricular lead with an active (screw) or passive (tine) fixation mechanism. High-sensitive Troponin T (hsTnT) was determined on the day of the procedure beforehand and on the following day. 326 Patients (median age (IQR) 75.0 (69.0–80.0) years, 64% male) from six international centers were randomized to receive ventricular leads with an active (n = 166) or passive (n = 160) fixation mechanism. Median (IQR) hsTnT levels increased by 0.009 (0.004–0.021) ng/ml in the group receiving screw-in ventricular leads and by 0.008 (0.003–0.030) ng/ml in the group receiving tined ventricular leads (n.s.). In conclusion pacemaker implantations are followed by a release of hsTnT. The choice between active or passive fixation ventricular leads does not have a significant influence on the extent of myocardial injury and the magnitude of hsTnT release.

## Introduction

Implantation of a pacemaker is the treatment of choice for many patients suffering from symptomatic bradycardia. Optimal ventricular lead fixation can be achieved with either active screw-in leads or passive tined leads. Both lead types can be used safely and provide reliable and adequate pacing thresholds in the long-term^1,2^. Complication rates are thought to be similar for both lead types. Therefore, the use of leads with active or passive fixation is very much dependent on the physician’s individual preference.

The implantation of a pacemaker is associated with myocardial micro damage^3–5^ which is typically quantified by measuring serum Troponin levels^6^. Active and passive fixation leads may both cause a Troponin release during the implantation procedure: in the case of active fixation leads due to the extension of the helix into the myocardium; and with passive fixation leads, due to entrapment within the ventricular trabecula during lead movement and the soft pressure used to eventually position the lead. It is unknown whether the lead fixation type affects the quantity of the implantation-related Troponin release. Faced with otherwise comparable lead performance of active and passive fixation leads, the lead type associated with the least implantation-related myocardial micro-damage may be favored.

Data on serum Troponin levels following pacemaker implantation is limited to the evaluation of absolute serum levels of standard Troponin I^3–5^ and lacks information on the currently preferred high sensitive Troponin tests. Furthermore, apart from one small, non-randomized study, these evaluations did not address the potential effect of different lead types^7^.

Therefore, the aim of the present trial is to compare the magnitude of the implantation-related serum high sensitive Troponin T (hsTnT) release in a larger number of patients receiving either an active fixation or a passive fixation ventricular lead.

## Methods

### Study Design

The PACMAN (Effect of **P**assive versus **Ac**tive fixation leads on the **Ma**gnitude of Troponi**n** Release after pacemaker implantation) trial is a prospective, randomized, multi-centre trial which recruited patients at six centres in Germany, Croatia and Japan (Deutsches Herzzentrum München, Klinikverbund Kempten-Oberallgäu, Dubrovnik General Hospital, University Hospital Dubrava, Tenri Hospital).

### Inclusion and exclusion

Patients with the indication for *de-novo* placement of a permanent single or dual chamber pacemaker (VVI or DDD pacemaker) were eligible for the study. Patients undergoing ventricular lead revisions could also be included provided that no attempt of lead extraction was performed.

Patients were excluded for any of the following reasons: presence of a temporary pacemaker, indication for cardiac resynchronization therapy or implantable cardioverter defibrillator placement. Patients who suffered from conditions that might be affiliated with an elevation or fluctuation of cardiac troponin levels were also excluded: patients in NYHA IV or in cardiogenic shock, patients scheduled for cardioversion within 24 hours after the procedure and patients with a history of pulmonary embolism, dialysis, heart surgery, acute coronary syndrome, revascularizations, cardioversions or ablations within four weeks of the pacemaker implantation in which hsTnT serum levels proved to be above the reference value. Additionally, patients with severe tricuspid regurgitation were not included. Finally, minors, pregnant women and patients who were not able to give informed consent were excluded.

### Randomization and study protocol

Following the provision of written informed consent and before pacemaker implantation, patients were randomized to receive either an active (screw-in) or passive (tined) ventricular lead. The randomization was conducted centrally in the German Heart Centre Munich in a 1:1 ratio stratified for single and dual chamber pacemaker implantations using varying block sizes. Study centres received the randomization schedule in sealed envelopes which were only to be opened directly before the procedure. The exact lead position in the ventricle was left to the discretion of the implanting physician. Atrial leads that had to be implanted used the active fixation technique. Leads had to be repositioned if sensitivity or pacing thresholds did not meet internal quality standards. Physicians aimed for a RA-Sensing ≥ 1.5 mV and a RV-Sensing ≥ 10 mV as well as pacing thresholds ≤ 1.0 V @ 0.5 ms for both leads. Cross-overs were documented and only allowed when the lead could not be implanted safely into the patient’s ventricle using several attempts with the fixation technique that the patient was randomized to. Data analysis was performed on an intention-to-treat basis.

Blood samples were taken on the morning of the implantation procedure and the following day. hsTnT was analysed using the “Roche Elecsys Troponin T high sensitive-Test” at each of the participating centres.

### Study endpoints

The primary endpoint was to quantify the myocardial micro-damage attributable to the implantation procedure. Therefore, the absolute difference of the serum hsTnT levels (Absolute ΔhsTnT) and relative difference (Relative ΔhsTnT = Absolute ΔhsTnT/baseline hsTnT) before and after pacemaker implantation was calculated for each patient.

The pre-specified secondary endpoint was to determine the proportion of patients before and after the procedure that showed hsTnT levels exceeding the 0.014 ng/ml cut-off value used for the diagnosis of acute myocardial infarction (AMI)^8–10^.

As additional endpoints that were not specified beforehand, the absolute and relative difference values of the serum hsTnT levels were characterized with respect to different lead diameters and single- vs. dual-chamber pacemakers and furthermore correlated with age.

### Ethics and trial registration

Ethics approval was obtained centrally at the Technische Universität München (Munich, Germany, project number 5272/12) and at each participating study centre as necessary. The trial was carried out under the regulations laid out in the Declaration of Helsinki and registered on ClinicalTrials.gov (Identifier NCT01897558, registration date 12/07/2013). All patients gave their written informed consent before participating in the study. Patient data was collected using pseudo-anonymized and standardized paper case report forms.

### Sample size calculation

In the absence of available data on hsTnT release following pacemaker implantation, historic data from 32 patients (16 patients with active and passive fixation ventricular leads each) with available hsTnT levels before and after pacemaker implantation was analyzed. The absolute ΔhsTnT levels were not normally distributed.

The null-hypothesis was formulated as follows: the probability of a randomly selected patient receiving an active fixation ventricular pacemaker lead showing a larger delta hsTnT than a randomly selected patient receiving a passive fixation ventricular pacemaker lead is 50%. The null hypothesis could be equally stated that the odds ratio (OR) in logistic regression analysis of ΔhsTnT vs. active/passive fixation equals 1. In our pilot cohort the actual probability was 59% (OR = 1.4). For sample size calculation it was assumed that a probability of 60% (OR = 1.5) in favor of any of the two groups, would be considered as clinically relevant. Based on two-tailed testing, a power of 80%, and a level of significance of α = 5%, a total of 262 patients had to be recruited (131 patients per group). Assuming a drop-out rate of 10%, at least 290 patients had to be enrolled (145 patients per group).

The study protocol required that a non-inferiority analysis would be conducted in case of failure to find statistical evidence for the superiority of one method compared to the other one. It was pre-defined that non-inferiority for clinical purposes would be concluded, if the lower limit of the 95% confidence interval for the area under the ROC curve (AUC) was larger than 0.45.

### Statistical methods

Statistical analyses of collected data were performed using data analysis software system Dell Statistica (version 12, 2015). Categorical data is presented by absolute and relative frequencies and compared using the Chi-Square or Fisher’s exact test where appropriate. Quantitative data distributions were tested for normality using Shapiro–Wilk’s test. As the majority of the data was non-normally distributed, all quantitative data is presented by median and the interquartile range (IQR). Comparisons of quantitative data were done using the Mann–Whitney U test or the Kruskal–Wallis ANOVA test, where appropriate. Correlational analyses were done by calculating Spearman rank order correlation coefficient. Statistical significance was determined at the level of 0.05 (two-tailed testing).

In order to test the null hypothesis, logistic regression was performed to establish correlation of ΔhsTnT with the type of procedure and the odds ratio was calculated. To determine non-inferiority, ROC analysis was performed both for absolute and relative ΔhsTnT as criteria variables for the two procedures.

### Data availability

The datasets generated and analysed during the current study are available from the corresponding author on reasonable request.

## Results

### Patient characteristics

**Figure 1:**
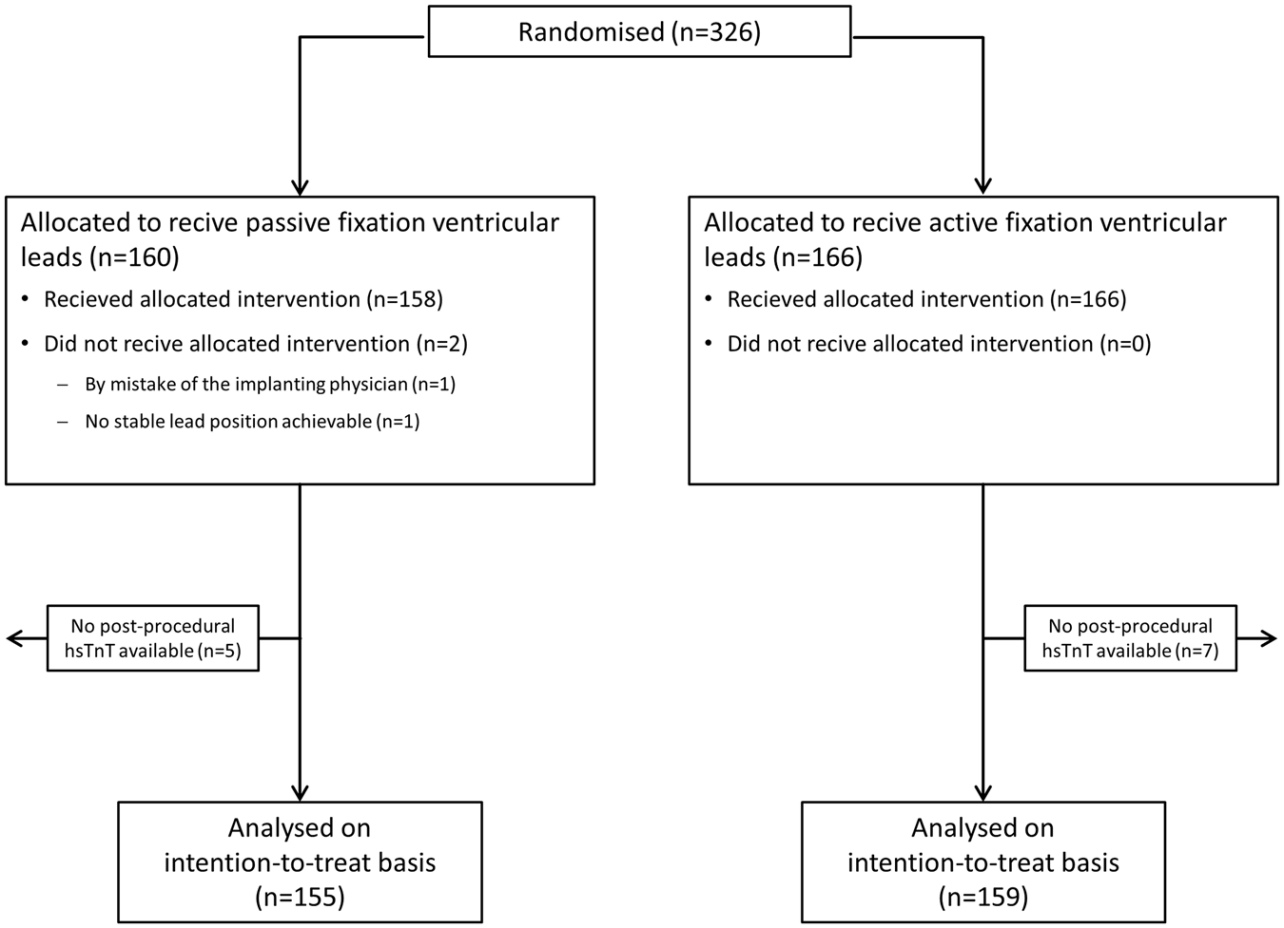
Study flow diagram.

Between April 2013 and December 2014 326 patients were included in the study, of whom 166 (50.9%) patients received a screw-in ventricular lead (active fixation) and 160 (49.1%) patients had a tined ventricular lead implanted (passive fixation). Patient inclusion was stopped after the coordinating center received notification about the inclusion of the 290^th^ patient. Patients who were scheduled for study participation at that time were allowed to enter the study if the implantation was performed within the next 4 weeks. Median age (IQR) in the total cohort was 75.0 (69.0–80.0) years, and by gender, the sample comprised 209 (64.1%) male patients. The study flow diagram, other baseline characteristics, indications for pacing and procedural data are provided in Figure 1, Tables 1, 2 and 3, respectively, alongside calculated levels of statistical significance (*p*-value) for comparison between the two cohorts (active and passive fixation). Cohorts did not differ significantly across baseline characteristics, indication for pacing, or procedural data, except for age (younger patients in the cohort with active fixation) and a trend towards a lower right ventricular-lead-diameter in the group with passive fixation leads. During the implantation procedure, cardioversion for supraventricular arrhythmias was required in two patients, one in the group with active fixation ventricular leads, one in the group with passive fixation ventricular leads. There were no other significant arrhythmias requiring cardioversion between the implantation procedure and the time that the second blood sample was taken.

**Table 1:** Baseline characteristics of the cohort and the two subgroups with respect to the ventricular fixation procedure.

	Total cohort		Active fixation		Passive fixation		*p*
	N	Value	N	Value	N	Value	
**Age** [years], *median (IQR)*	326	75 (69–80)	166	74 (69–79)	160	76 (71–81)	0.025
**Gender** - male, *n (%)*	326	209 (64.1%)	103 (62.0%)		106 (66.3%)		0.429
**BMI** [kg/m^2^], *median (IQR)*	313	26.8 (24.2–29.1)	160	26.5 (24.1–28.8)	153	26.8 (24.5–29.3)	0.363
**Obesity** (BMI ≥30), *n (%)*	311	62 (19.9%)	28 (17.6%)		34 (22.4%)		0.295
**Coronary artery disease**, *n (%)*	326	115 (35.3%)	61 (36.7%)		54 (33.8%)		0.571
**Valvular disease**, *n (%)*	326	93 (28.5%)	40 (24.1%)		53 (33.1%)		0.071
**Hypertension**, *n (%)*	326	287 (88.0%)	150 (90.4%)		137 (85.6%)		0.188
**Hyperlipidemia**, *n (%)*	326	187 (57.4%)	99 (59.6%)		88 (55.0%)		0.397
**Diabetes mellitus**, *n (%)*	323	91 (28.2%)	39 (23.6%)		52 (32.9%)		0.064
**Active smoker**, *n (%)*	324	59 (18.2%)	29 (17.7%)		30 (18.8%)		0.803
**PAOD**, *n (%)*	324	41 (12.7%)	21 (12.8%)		20 (12.5%)		0.934
**LV-EF** [%], *median (IQR)*	291	60 (53–64)	148	60 (54–65)	143	60 (52–64)	0.209
**LV-EF** <55%, *n (%)*	291	80 (27.5%)	38 (25.7%)		42 (29.4%)		0.481
**Baseline Creatinine** [mg/dl], *median (IQR)*	325	1.08 (0.89–1.30)	166	1.07 (0.89–1.24)	159	1.10 (0.89–1.39)	0.444
**Baseline hsTnT[ng/ml]**, *median (IQR)*	326	0.014 (0.009–0.024)	166	0.013 (0.008–0.023)	160	0.016 (0.010–0.024)	0.143
**Post-proc hsTnT** [ng/ml], *median (IQR)*	314	0.026 (0.016–0.041)	159	0.026 (0.016–0.041)	155	0.026 (0.016–0.043)	0.954
**Absolute Δ hsTnT** [ng/ml], *median (IQR)*	314	0.009 (0.004–0.020)	159	0.009 (0.004–0.021)	155	0.008 (0.003–0.020)	0.506
**Relative Δ hsTnT** [%]	314	60.6 (20.4–150.8)	159	75.0 (26.1–177.8)	155	52.6 (17.7–132.8)	0.078

BMI = Body Mass Index, PAOD = Peripheral Artery Occlusive Disease, LV-EF = Left Ventricular Ejection Fraction.

**Table 2:** Indications for pacing.

	Total cohort	Active fixation	Passive fixation	*p*
Atrioventricular Block, *n (%)*	131 (40.2%)	65 (39.2%)	66 (41.3%)	0.373
Sick-Sinus-Syndrome, *n (%)*	103 (31.6%)	58 (34.9%)	45 (28.1%)	
Atrial fibrillation with bradycardia, *n (%)*	73 (22.4%)	32 (19.3%)	41 (25.6%)	
Others, *n (%)*	19 (5.8%)	11 (6.6%)	8 (5.0%)

**Table 3:** Procedural data.

	Total cohort		Active fixation		Passive fixation		*p*
	N	Value	N	Value	N	Value	
**Single Chamber Pacemaker**, *n (%)*	326	124 (38.0%)		61 (36.7%)		63 (39.4%)	0.625
**Dual Chamber Pacemaker**, *n (%)*	326	202 (62.0%)		105 (63.3%)		97 (60.6%)	
**RA lead diameter [Fr]**, *median (IQR)*	202	6.0 (5.6–6.0)	105	6.0 (5.6–6.0)	97	6.0 (5.6–6.0)	0.159
**RV lead diameter [Fr]**, median (IQR)	326	6.0 (6.0–6.0)	166	6.0 (6.0–6.0)	160	6.0 (5.3–6.0)	0.002
**RA lead diameter** [Fr]							
<6.0, *n (%)*		90 (44.6%)		44 (41.9%)		46 (47.4%)	0.085
=6.0, *n (%)*		84 (41.6%)		41 (39.0%)		43 (44.3%)	
>6.0, *n (%)*		28 (13.9%)		20 (19.0%)		8 (8.2%)	
**RV lead diamete**r [Fr]							
<6.0, *n (%)*		69 (21.2%)		19 (11.4%)		50 (31.3%)	<0.001
=6.0, *n (%)*		201 (61.7%)		116 (69.9%)		85 (53.1%)	
>6.0, *n (%)*		56 (17.2%)		31 (18.7%)		25 (15.6%)	
**Right-Atrial Lead Locations** (Attempts), *median (IQR)*	190	2 (1–4)	98	2 (1–3)	92	2 (1–4)	0.225
**Right-Atrial-Screw-Ins** (Attempts), *median (IQR)*	190	1 (1–2)	98	1 (1–2)	92	1 (1–2)	0.730
**Right-Ventricular Lead Locations** (Attempts), *median (IQR)*	312	3 (2–5)	160	3 (2–5)	152	2 (2–4)	0.169
**Right-Ventricular-Screw-Ins** (Attempts), *median (IQR)*	314	1 (0–1)	160	1 (1–2)	154	0 (0–0)	<0.001
**Fluoroscopy: Dose area product** [cGycm²], *median (IQR)*	146	270 (131–610)	76	309 (132–723)	70	261 (125–503)	0.484
**Procedure time [min]**, *median (IQR)*	313	55 (38–70)	159	55 (40–72)	154	53 (36–68)	0.152
**Time after procedure until 2nd blood sample was taken** [hours], *median (IQR)*	116	17.9 (15.7–20.0)	60	17.8 (15.3–19.8)	56	18.4 (16.3–20.0)	0.495

RA = Right-atrial, RV = Right-ventricular.

### Primary Endpoint

**Figure 2:**
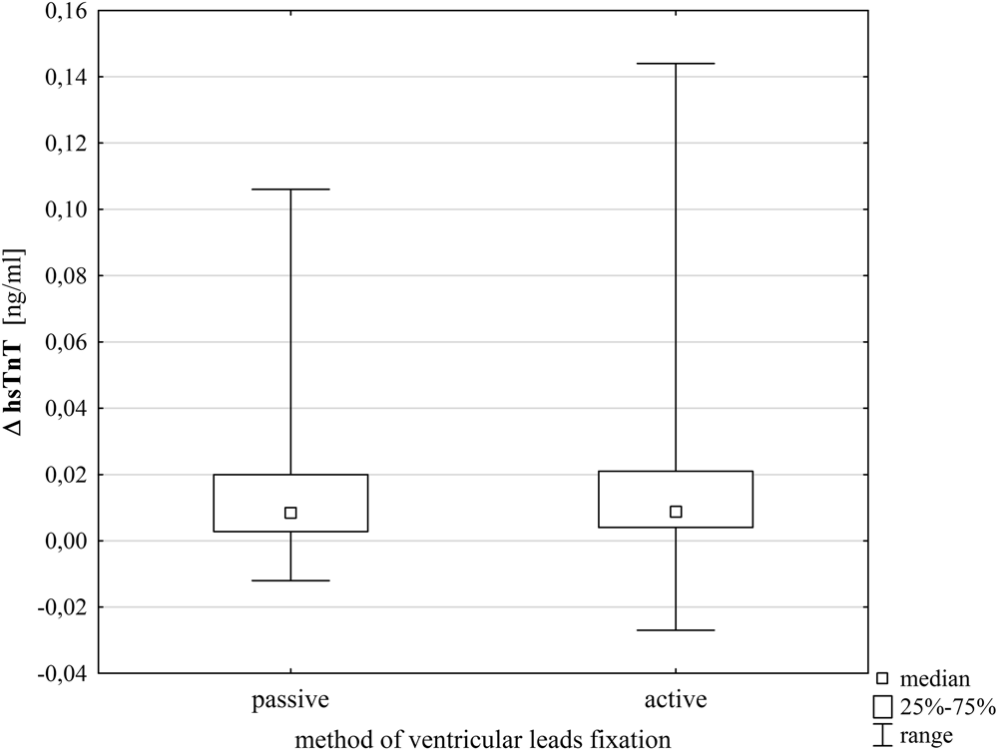
Absolute difference of troponin levels (ΔhsTnT) in the groups with active and passive fixation ventricular leads (a single outlier in the group with active fixation ventricular leads with ΔhsTnT = 0.297 ng/ml is excluded). hsTnT = High-sensitive Troponin T.

We determined baseline hsTnT values and post-procedural hsTnT values, and calculated absolute ΔhsTnT and relative ΔhsTnT for 314 patients. Seven patients in the group with active fixation ventricular leads and five in the group with passive fixation ventricular leads had to be excluded from the analysis as their post-procedural hsTnT levels were missing. Overall postoperative hsTnT values were significantly higher (postoperative hsTnT median 0.026 (0.016–0.041) ng/ml vs. baseline hsTnT median 0.014 (0.009–0.024) ng/ml, Wilcoxon matched pairs test, p < 0.001). The absolute ΔhsTnT did not differ significantly between the groups with active and passive fixation leads (p = 0.506), while the relative ΔhsTnT showed marginally significant difference (p = 0.078), as presented in Table 1. The absolute ΔhsTnT was higher in the patients receiving dual-chamber pacemakers than in the patients receiving single-chamber pacemakers (DDD (n = 194): 0,011 (IQR 0,006–0,025), VVI (n = 120): 0,005 (IQR 0,002–0,011) ng/ml, p =  < 0.001). There was however no significant difference in absolute ΔhsTnT between active and passive ventricular lead fixation when stratifying between single- and dual-chamber pacemakers (VVI: 0.004 (0.001–0.010) ng/ml for passive fixation and 0.006 (0.003–0.012) ng/ml for active fixation ventricular leads (p = 0.123); DDD: 0.013 (0.006–0.025) ng/ml for passive fixation and 0.010 (0.006–0.027) ng/ml for active fixation ventricular leads (p = 0.794)). Patient’s differences in Troponin levels (ΔhsTnT) from groups with active and passive fixation are shown in Fig. 2.

The probability of a randomly selected patient receiving an active fixation ventricular pacemaker lead showing a larger ΔhsTnT than a randomly selected patient receiving a passive fixation ventricular pacemaker lead was calculated to be 53.5% (95% CI:26.5–80.5). The same type of test was done by calculating the odds ratio with logistic regression (ΔhsTnT - active/passive procedure) giving an OR = 1.15 (95% CI: 0.36–4.13), being non-significant. Both results suggest that the data did not satisfy the pre-specified margin of clinical relevance (probability of 60% or OR = 1.5).

To ascertain non-inferiority, ROC analysis was performed both for the absolute and relative ΔhsTnT as criteria variables for the two procedures. The results are presented in supplementary Table S.1. As the results suggest, in both cases non-inferiority could be concluded, since in both cases the lower limit of 95% CI for AUC was >0.45.

### Secondary Endpoint

The secondary endpoint was to determine the proportion of patients with hsTnT values that exceeded the upper reference limit for the diagnosis of AMI (hsTnT > 0.014 ng/ml), both before and after the procedure. Data is shown in Table 4.

**Table 4:** Occurrence of elevated hsTnT levels with respect to cut-off value (0.014 ng/ml) for AMI diagnosis.

Baseline hsTnT [ng/ml]	Total cohort		Active fixation		Passive fixation	
	Post-proc. hsTnT [ng/ml]		Post-proc. hsTnT [ng/ml]		Post-proc. hsTnT [ng/ml]	
	≤0.014	>0.014	≤0.014	>0.014	≤0.014	>0.014
**≤0.014**, *n(%)*	63 (39%)	98 (61%)	32 (36%)	56 (64%)	31 (42%)	42 (58%)
**>0.014**, *n(%)*	1 (1%)	152 (99%)	0 (0%)	71 (100%)	1 (1%)	81 (99%)
Sum, *n(%)*	64 (20%)	250 (80%)	32 (20%)	127 (80%)	32 (21%)	123 (79%)

hsTnT = High-sensitive Troponin T.

Before the procedure, 153 out of 314 (48.7%) patients in the total cohort showed a baseline hsTnT level that exceeded the cut-off for AMI diagnosis. There was no difference between the groups that received active or passive fixation; 71 patients were in the group with active fixation (44.7% of the active fixation group) and 82 patients were in the group with passive fixation (52.9% of the passive fixation group), indicating no statistically significant difference (Fisher exact test, p = 0.089). After the implantation, the number of patients showing hsTnT > 0.014 ng/ml rose to 250 (79.6% of the total cohort). The number of patients with a chang in hsTnT levels (from baseline hsTnT ≤ 0.014 to post procedural hsTnT > 0.014) was 98 (31.2% of the total cohort) and did not differ in two groups with respect to the procedure (56/159 = 35.2% in active fixation group vs. 42/155 = 27.1% in the passive fixation group, p = 0.121).

### Relationship between Ventricular Lead Diameter and hsTnT release

Patients in the groups with active and passive fixation ventricular leads differed significantly with respect to ventricular lead diameters. As an exploratory analysis, to rule out an influence of the lead diameter on our result, we analyzed the association of the ventricular lead diameter with both absolute and relative ΔhsTnT values. No correlation was detected between the ventricular lead diameter and ΔhsTnT values for the total cohort (Spearman rank r = 0.02 for absolute and r = 0.05 for relative ΔhsTnT). Interestingly, the results changed for the groups by fixation procedure-type; namely, in the group with active fixation the correlation was positive and significant (Spearman rank r = 0.22 or absolute and r = 0.27 for relative ΔhsTnT) and negative, but insignificant (Spearman rank r = −0.21 for absolute and r = −0.13 for relative ΔhsTnT) in the group with passive fixation.

The cohort was stratified according to ventricular lead diameter of 6 Fr. The patients were divided into three groups: the first with a ventricular lead diameter < 6 Fr (N = 65), the second with a diameter = 6 Fr (N = 196) and the third with a diameter > 6 Fr (N = 53). Results are presented in supplementary Table S2. We could not find a significant difference in either absolute ΔhsTnT levels (Kruskal-Wallis ANOVA, p = 0.080) or in relative ΔhsTnT levels (Kruskal-Wallis ANOVA, p = 0.086) between the three groups. However, when the same analysis was performed with respect to the procedure applied (active or passive fixation), the results showed significant differences (both for active and passive fixation procedure and for absolute and relative ΔhsTnT). Interestingly, the trend appeared to be different in the two cohorts; in the group receiving active fixation, the largest significant Troponin release was detected in the group with RVL diameter > 6 Fr (Kruskal-Wallis ANOVA, p = 0.030 for absolute ΔhsTnT and p = 0.005 for relative ΔhsTnT), conversely, in the group receiving passive fixation the largest Troponin release was detected in the group with RVL diameter < 6 Fr (Kruskal-Wallis ANOVA, p = 0.070 for absolute ΔhsTnT and p = 0.036 for relative ΔhsTnT). These results simultaneously explain the significantly larger absolute and relative ΔhsTnT values in the group with RVL diameter < 6 Fr for the passive fixation procedure relative to the active procedure.

### Relationship between serum hsTnT values and age

Despite the randomization, the passive fixation group appeared to be significantly older (Table 1). In order to determine whether the observed results on serum Troponin levels could be confounded by the patient’s age, we performed correlation analysis for all the serum Troponin levels (measured and calculated) with age. As can be seen from the data presented in supplementary Table S.3, baseline and post-procedural Troponin levels correlate positively and significantly with age; however, the differences in hs Troponin levels (both absolute and relative) are not correlated with age whatsoever, thus confirming that patient age did not confound the study results.

## Discussion

### Relationship of ventricular fixation mechanism and ΔhsTnT

Data on the potential influence of the lead type on myocardial micro-damage is scarce. In our randomized, prospective study consisting of 326 patients we could not find statistically significant differences in the peri-procedural hsTnT release when comparing active and passive fixation ventricular leads.

Similar results have been obtained from sub-group analyses of small, non-randomized trials of implantable cardioverter defibrillator placement^11,12^. However, it is questionable whether the results of these trials can be assumed to be applicable to pacemaker leads. Implantable cardioverter defibrillator leads have a significant different geometry which may impact trauma and Troponin release. Additionally, during implantable cardioverter defibrillator placement, defibrillation threshold tests were performed which are known to affect serum Troponin levels post implantation^11,13–15^.

The only available data on Troponin (but not hs Troponin) release in relation to different pacemaker leads is from a study by Martignani *et al*.^7^. This study suggests that the implantation of screw-in leads causes higher levels of Troponin I than the use of tined leads. This is contradictory to our own finding that passive leads are as traumatic for the myocardium during implantation as active leads. However, the trial by Martignani *et al*. was limited by the relatively small number of patients and lack of randomization. Additionally, the applied Troponin I assay did not allow for the detection of subtle changes in the Troponin release which may have further influenced the results. Considering the superiority of high sensitive Troponin assays in detecting myocardial damage in general^16^, we believe that the assessment of damage at a micro-level is optimally achieved with high sensitive Troponin assays as used in our trial.

The observation of serum Troponin elevation following pacemaker implantation is consistent with previous findings^3–5,7^. However, available data from small studies focused on the determination of serum Troponin I rather than serum Troponin T levels and failed to address high sensitive methods for Troponin detection. Adopting a widely used high sensitive Troponin T assay, in our study the median release of Troponin T attributable to a pacemaker implantation was determined to be 0.009 ng/ml or 60.6% of the baseline value.

The magnitude of Troponin release has been linked to the patient’s short- and long-term outcome in several clinical circumstances^17–23^ and even small increases in hsTnT seem to be relevant for the patient’s prognosis^24^. This data may not necessarily be applicable to the pacemaker implantation procedure. Nevertheless, it may be prudent to develop implantation strategies which limit the Troponin release in this setting. In our study, the type of lead fixation did not have a significant influence on the release of hsTnT. There was however a relationship between the choice of either a single- or dual-chamber-pacemaker and the amount of hsTnT release. Even though this seems intuitive and has already been described before in ICDs as well as in pacemakers^5,11^, further trials are warranted to look into this effect as our study was not powered for this comparison.

### Proportion of patients with hsTnT values above the level for diagnosis of AMI

As a secondary endpoint we determined the percentage of patients with hsTnT values above the level suggesting myocardial micro-damage, e.g. the percentage of patients exceeding the reference value used for the diagnosis of AMI (0.014 ng/dl). There was no statistically significant difference between the groups assigned to receive an active or passive fixation ventricular lead. Interestingly, at baseline approximately 50% of the patients showed hsTnT serum levels above the reference value for AMI. This was despite the absence of clinical signs of AMI or ECG changes suggestive for acute coronary ischemia. Therefore, it is assumed that bradycardia-associated reduction in cardiac index and co-morbidities such as renal failure had mainly contributed to the elevated values.

Following pacemaker insertion in both groups approximately 80% of patients exceeded the hsTnT reference value of AMI without presenting symptoms suggestive of acute coronary ischemia. This finding corroborates the limitations of the use of hsTnT serum concentration after a pacemaker implantation in the diagnosis of AMI. As the ECG may not also be suitable for the diagnosis of AMI (paced QRS, pacing induced ST segment alterations), a judgment based on clinical symptoms is of paramount importance when AMI is suspected directly after pacemaker insertion. This observation is congruent with previous studies evaluating Troponin I after pacemaker implantation^4,5,7^. Using standard assays for Troponin detection in these studies, 2% to 39% of patients post-procedurally exceeded the respective reference values used at the time of study conduct. Using a high sensitive assay, in our trial even a larger proportion of patients was affected by this procedure-induced elevation of serum Troponin T above the current reference value.

### Relationship between Ventricular Lead Diameter and ΔhsTnT

A small study previously reported differences in lead diameters affecting serum Troponin levels after pacemaker implantation; with smaller lead diameters resulting in lower post-procedural serum Troponin levels^5^. In our study, leads with a tip diameter less or equal 6 Fr were significantly more frequently found in the group with passive fixation leads. Given the relatively uniform lead tip diameters ranging between 5.3 and 7.2 Fr. we do not believe that the slight imbalance in the baseline characteristics had significantly influenced the overall results of our study. Unlike a previous study^5^, in sub-group analysis we could not reproduce the result of smaller lead tip diameters being linked to lower serum hsTnT or delta hsTnT post-procedurally. As our study was not powered for this analysis however, further studies are warranted to evaluate the impact of the lead size and other potential contributors such as individual implantation techniques on myocardial micro-damage resulting from the pacemaker implantation procedure.

### Relationship between age and ΔhsTnT

Despite randomization, patients assigned to the group of passive ventricular fixation leads were slightly, but significantly older. It is known that serum levels of Troponin T in unselected patients increases with age^8,25–29^. However, it is unlikely that this difference would remain significant following adjustment for co-morbidities^25,26,28^. As the primary endpoint of our study was an intra-individual comparison of serum hsTnT levels before and after pacemaker implantation, we showed that in spite of existing correlation of serum hsTnT levels with age, there was no measurable association of intra-individual differences of hsTnT values with patients’ age. Therefore, we concluded that the differences in age between the two treatment groups had no influence on the study results.

### Limitation

Although serum Troponin levels have been shown to be associated with the long-term outcome of patients in different clinical settings^17–24^ it is not known whether there is a similar association in the context of pacemaker implantation. In the absence of data evaluating the effect of Troponin release on the long-term prognosis of patients following pacemaker implantation, the limitation of myocardial damage during pacemaker implantation seems an intuitive strategy. For this acute aim, the choice of the fixation type of the lead does not seem to play a significant role.

The PACMAN trial did not evaluate cardiac biomarkers pointing towards myocardial “macro-damage” such as serum levels of overall creatine kinase (CK) or CK-MB. Given the fact that myocardial micro-damage did not differ between the two groups it seems highly unlikely that differences on a macro-level of myocardial injury are present.

## Conclusion

The implantation of a permanent pacemaker lead is associated with an elevation of serum high sensitive Troponin T level, which in the majority of patients exceeds the diagnostic reference value for acute myocardial infarction. High sensitive Troponin T release attributable to the implantation procedure did not differ significantly between active versus passive fixation ventricular pacemaker leads.

## Electronic supplementary material

PACMAN Study Protocol

PACMAN CONSORT 2010 Checklist

Supplementary Tables
