# Supplementary material for: Pacemaker Implantation Associated Myocardial Micro-Damage: A Randomised Comparison between Active and Passive Fixation Leads
Source: Sci Rep. 2018 Mar 20;8:4870. doi: 10.1038/s41598-018-23209-5 (PMC5861101; doi:10.1038/s41598-018-23209-5)
Supplement: Supplementary file 1 — PACMAN Study Protocol [file 41598_2018_23209_MOESM1_ESM.pdf]

Deutsches Herzzentrum München
Lazarettstraße 36
80636 München
Germany
Tel +49 89 1218-2020

CLINICAL STUDY PROTOCOL

Effect of **P**assive versus **Ac**tive fixation leads on the **Ma**gnitude of Troponi**n** Release after pacemaker implantation

PACMAN Trial

Academic investigators:

PD Dr. med. Christof Kolb
Deutsches Herzzentrum München, Klinik für Herz- und Kreislauferkrankungen, München, Germany

Dr. Haitham Badran
Ain Shams University, Cardiology Department, Cairo, Egypt

1. Background:

Implantation of a pacemaker is the treatment of choice for bradyarrhythmias. The short- and long-term success of permanent pacing is critically dependent on a stable position of the electrode. Historically, the first pacing electrodes were lacking mechanisms for fixation and therefore were associated with an unacceptably high risk of dislodgement. With the development of passive and active myocardial fixation mechanisms for electrodes the need for lead repositioning has been dramatically reduced (1).

Passive fixation leads are typically equipped with small plastic tines that entrap within the trabeculae of the right heart chambers immediately upon correct positioning of the lead. For active fixation leads different fixation mechanisms (screws, barbs or hooks) have been developed, but active screws represent the most frequently used type (2,3,4).

Both lead types encounter their own specific advantages and disadvantages.

Passive fixation electrodes show less pronounced electric injury currents during implantation and therefore judgement of the appropriate lead position and the pacing and sensing capabilities are facilitated. They can be easily used in a typical right ventricular apical lead position but are not deemed to be ideal for alternate pacing locations such as the right ventricular mid septum, the outflow tract or the his bundle region. Passive fixation leads are rapidly covered by fibrous tissue, making later removal of the lead by simple traction more difficult.

Active fixation leads with an extendable and retractable screw design are widely used for their ease of implantation irrespective of the final lead position in the heart. Advantages during lead revisions include the ability to retract the screw even years after implantation and thus allow easier extraction procedures (3). Disadvantages of active fixation leads mainly include a trend to slightly higher rates of perforation during implantation (5). Additionally, there is a belief that active fixation electrodes may be more aggressive to the myocardium during implantation and that they could cause a more pronounced myocardial micro-damage on implantation.

Because the stimulation thresholds of active and passive fixation leads tend to be similar, and differences in the rate of lead dislodgement and perforation are marginal (trend towards superiority of active fixation leads in the first and passive fixation leads for the latter), the choice of an active or a passive ventricular fixation electrode is largely a matter of personal

preference of the implanting physician (4). This is especially true for the right ventricular apical lead position which encounters >90% of ventricular lead positions.

Bearing in mind the equal performance of both lead types, the ability to implant the electrode as atraumatic as possible has come into the focus for electrode selection. Transvenous insertion of either passive or active endocardial leads for permanent pacing is accompanied by troponin elevation compatible with myocardial damage, secondary to the direct myocardial trauma elicited by pacing leads. Published data mostly report troponin elevations within or very close to the range of “minimal myocardial damage” (6,7,8) but usually did not exceed 3 ng/ml (9). These cardiac troponin-I (CTN-I) elevations occurred within 6 h after implantation (6).

Although active fixation leads are thought to be potentially more traumatic and therefore may be correlated with a higher troponin release, no randomized trial has been conducted to evaluate myocardial micro-damage with respect to different electrode types.

Therefore the primary aim of the PACMAN trial is to investigate whether ventricular active or passive fixation electrodes are associated with a different amount of myocardial micro-damage which is reflected by potentially different levels of high-sensitivity troponin release after pacemaker implantation.

2. Hypothesis:

The implantation of a pacemaker system is associated with an increase in the plasma concentrations of the high-sensitivity troponin T which serves as a surrogate for myocardial damage. The increase in plasma concentrations of high-sensitivity troponin T is not related to the use of an active or a passive fixation of the ventricular lead.

3. Study design:

The PACMAN trial is designed as a prospective randomised, multi-centre, international study.

4. Inclusion and exclusion criteria:

Inclusion

- Indication of de-novo (single or dual chamber) pacemaker implantation according to current national or international guidelines

Or

- Additional ventricular lead insertion on the occasion of lead revisions with no explantation attempt for abandoned leads

Exclusion

- Cardiac resynchronisation therapy or AAI pacing
- Revision (except for adding a ventricular lead to pre-existing leads)
- Temporary pacing
- ICD indication
- Right ventricular pacing aimed from the mid-septum, outflow tract or his bundle region
- Severe tricuspid regurgitation requiring active fixation leads
- Planned cardioversion during pacemaker implantation or within 24 hours thereafter
- NYHA IV
- (Cardiogenic) shock
- Heart Surgery, acute coronary syndrome, myocardial infarction, any revascularisation, cardioversion or ablation procedure within the last 4 weeks with elevated base line high-sensitivity troponin levels.
- Pulmonary embolism, stroke, dialysis within the last 4 weeks
- Patient not available for follow-up
- Patient's inability to consent or refusal to consent
- Age <18 years, pregnancy

5. Study procedure:

After obtaining written informed consent to the pacemaker implantation and the study participation the baseline high-sensitivity troponin T plasma concentration will be determined. This will be done from a previously taken blood sample provided for standard clinical tests if the sample is not older than 24 hours. If no such sample is available or if retrospective analysis is not possible an additional blood sample is required. Prior to pacemaker implantation the patient will be randomly assigned to receive a CE-marketed, commercially available, ventricular passive or active fixation electrode. Randomisation will be stratified for single- and dual-chamber pacing and for centres in a 1:1 ratio between active fixation and passive fixation leads for right ventricular pacing; for atrial leads active fixation will be used. The randomisation will be performed in varying blocks for each of stratify and will be centrally generated in the Deutsche Herzzentrum München. The participating centres will be provided with sealed envelopes containing the randomisation information. During pacemaker

implantation all necessary – and clinically reasonable - efforts should be made to adhere to the randomised lead type. Cross-overs (to avoid unsuccessful pacemaker implantations) will be allowed on the basis of the individual judgement of the implanter. In case of cross-over, the primary endpoint will be evaluated on an intention-to-treat basis. After pacemaker implantation, the patient will be followed according to standard clinical practice. Standard clinical practice includes the drawing of blood samples post-implantation (usually the next morning). For study reasons this should be done between 6 and 24 hours after pacemaker implantation. On this occasion, additionally to the routine parameters, high-sensitivity troponin T plasma concentration will be determined. The patients will be followed for three months with respect to lead performance, necessity of lead revision and mortality.

6. Endpoints:

Primary endpoint:

The primary endpoint is based on the comparison of the increase of high-sensitivity troponin T plasma concentrations (measured with Roche hs-TropT assay; difference between post-implantation and pre-implantation concentrations) between patients receiving a passive fixation or an active fixation ventricular electrode.

Secondary endpoints:

- Proportion of patients reaching a high-sensitivity troponin T level >0.014 ng/dl (formally acute coronary syndrome)
- High-sensitivity troponin T plasma concentrations level post-implantation in relation to procedure duration and fluoroscopy time, number of mapping sites during implantation, ejection fraction, serum-creatinine, single- or dual chamber pacemaker implantation, age, ischemic versus non-ischemic heart disease, and lead tip size
- Pacing and sensing performance of the ventricular leads after 3 months
- Lead-related morbidity (dislodgement, perforation) and mortality within three months

7. Study related risks:

In both arms CE-marketed and commercially available electrodes will be used. There is no consensus which lead type (active or passive fixation) is the better one. Lead selection in clinical practise is based on personal preference of the implanter. As only centres experienced in the implantation of both lead types are accepted for the trial and circumstances which

require a specific fixation mechanism of the electrode are defined as exclusion criteria, there is no attributable risk to study participation of the patient and the randomisation procedure.

For study reasons additional laboratory analyses (high-sensitivity troponin T plasma concentration) will be performed. These analyses can - in the vast majority of cases - be made from samples that are drawn routinely. In few patients an additional drawing of blood (2 ml) will be required. The patient will not be charged for these additional analyses.

Data acquisition and handling is pseudo-anonymous and only the principle investigator of each contributing centre will be able to allocate acquired data to the name of the patient.

Thus, there is neither potential risk for the patients in participating in the study nor is there any benefit for the participating individual.

8. Setting of the study

In order to include a reasonable number of patients at each of the centres, only high volume centres of pacemaker implantation will be invited to participate. These centres have to be experienced in the implantation of active and passive fixation ventricular leads.

Planned investigational sites are by now

- Deutsches Herzzentrum München, Klinik für Herz- und Kreislauferkrankungen, München, Germany
- Ain Shams University, Cardiology Department, Cairo, Egypt
- Klinikum rechts der Isar, 1. Medizinische Klinik, München, Germany
- Universitätsklinikum Dubrava, Abteilung für Kardiologie, Zagreb
- Krankenhaus Landshut-Achdorf, Medizinische Klinik I, Landshut, Germany
- Henry Dunant Hospital, Department of Cardiology, Athen, Greece
- S. Giovanni Calibita Fatebenefratelli Hospital, Department of Cardiology, Isola Tiberina, Rome, Italy

9. Statistics

Because there is no data available on high-sensitivity troponin T release after pacemaker implantation, historic data from patients supplied with pacemakers in the German Heart Centre 2011 were used for sample size calculation. For this, data of the most recent 30 patients (most recent 15 patients with active fixation ventricular leads and most recent 15 patients with passive fixation ventricular leads) with available high-sensitivity troponin T levels before and after pacemaker implantation were analysed. They did not differ relevantly in their baseline

characteristics and the distribution of high-sensitivity troponin T plasma concentrations is shown in the following table.

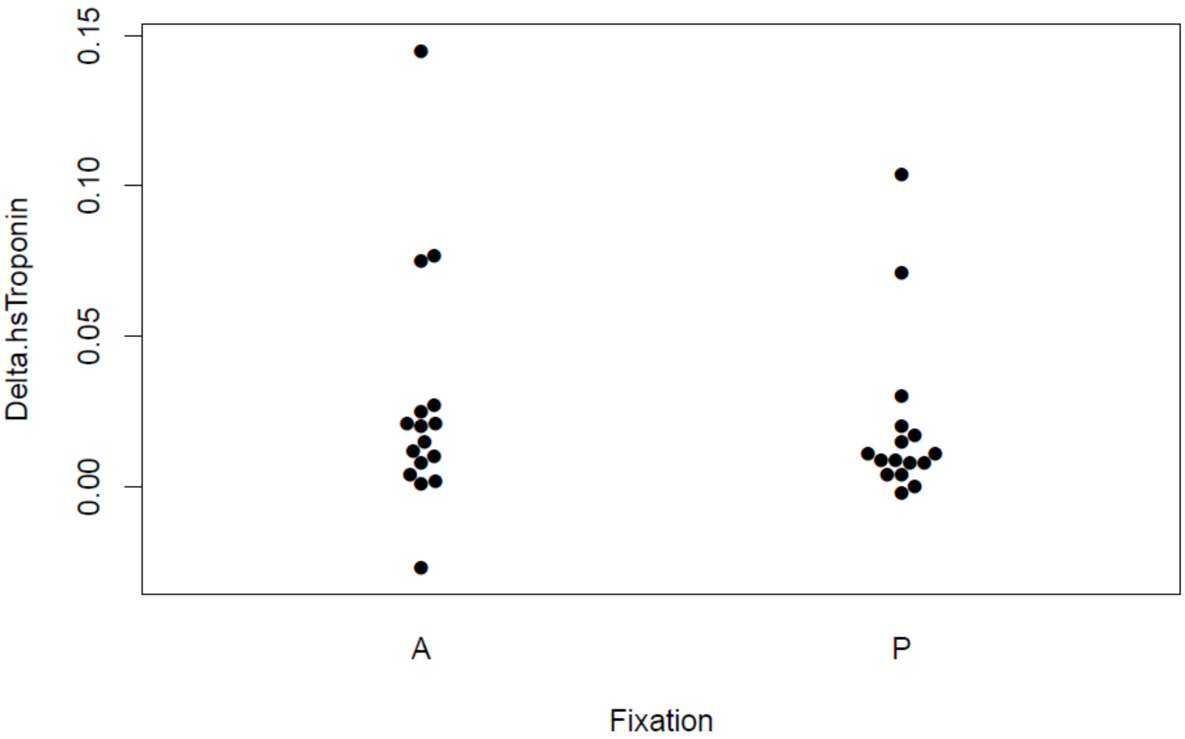

A=active fixation; P=passive fixation;
delta.hsTroponin refers to change plasma concentrations measured in ng/ml

The distribution is non-normal and due to large variations in the values including negative increases of plasma concentration after pacemaker implantation a non-parametric test (Mann-Whitney U Test) is the most appropriate approach to sample size estimation.

Null hypothesis: The probability that a randomly selected patient of the study cohort who received an active fixation ventricular pacemaker electrode shows a larger increase of high-sensitivity troponin T after implantation than a randomly selected patient who received a passive fixation ventricular pacemaker lead is 50 %. H_0: P(A > P) = P(P > A) = 50% .

In the cohort of historic patients from the German Heart Centre the above mentioned probability was P(A > P) = 59%. For sample size calculation it was assumed that an effect of P(A > P) = 60% or P(P > A) = 60% would determine the least difference that would be considered as clinically relevant..

Assuming that there is a relevant difference between both groups, a total of 262 patients need to be recruited (131 patients per group) to find a significant effect with a probability (=power) of at least 80% on a two-sided level of significance of α=5% (10).

Assuming a drop-out rate of 10% for this acute study, a total of 290 patients (145 patients per group) will be enrolled in the study.

<u>Assessment of non-inferiority:</u>

If the study fails to find statistical evidence for superiority of one method compared to the other, non-inferiority will be concluded, if the lower limit of the 95% confidence interval for the area under the ROC curve (AUC) is greater than 0.45. The AUC estimates the probability that the change in high-sensitivity troponin T plasma concentration is greater in a randomly selected patient treated with one method compared to a random sample from patients treated with the other method.

10. Study start:

Planned after ethics committee approval 03/2012

11. Principle investigators:

PD Dr. med. Christof Kolb, Klinik für Herz- und Kreislauferkrankungen, Deutsches Herzzentrum München

Dr. Haitham Badran, Ain Shams University, Cardiology Department, Cairo, Egypt

12. References

1. Holmes DR, Nissen RG, Maloney JD, et al. Transvenous tined electrode systems an approach to acute dislodgment . Mayo Clin. Proc. 1979; 54: 219-222.
2. Markewitz A. [2008 Annual Report of the German Pacemaker Registry: Cardiac Pacemaker Specialty Group and BQS Federal Quality Assurance Office gGmbH (manager: Dr. C. Veit), Düsseldorf]. Herzschrittmacherther Elektrophysiol 2010;21:256-84.
3. Bispin HJ, Kreuzer J, Birkenheir H. Three – year clinical experience with a new endocardial screw in lead with introduction protection for the use in the atrium and ventricle. Pacing Clin Electrophysiol 1980; 3:424-435
4. Pehrsson SK, Bergdahl L, Svane B, Early and late efficacy of three types of transvenous atrial leads. Pacing Clin Electrophysiol 1984; 7:195-202.
5. Stefanidis AS, Margos PN, Kotsakis AA, Papasteriadis EG. Three-dimensional echocardiographic documentation of pacemaker lead perforation presenting as acute pericarditis. Hellenic J Cardiol. 2009; 50: 335-337
6. Boos CJ, Gough S, Wheather M, Medbak S, More R. Effects of transvenous pacing on cardiac troponin release. Pacing Clin Electrophysiol. 2004; 27: 1264-1268.

7. Martignani C, Diemberger I, Biffi M, et al. Troponin I rise after pacemaker implantation at the time of "universal definition of myocardial infarction". Am J Cardiol. 2009; 103: 1061-1065.
8. Nikolaou NI, Spanodimos SG, Tsaglis EP, et al. Biochemical evidence of cardiac damage following transvenous implantation of a permanent antibradycardia pacemaker lead. Pacing Clin Electrophysiol 2005; 28: 1174-1181.
9. Nikolaou NI, Christou AP, Spanodimos SG et al. Marked Troponin elevation after implantation of a permanent antibradycardia pacemaker. Hell J Cardiol 2011;52:489-92.
10. Noether GE. Sample size determination for some common nonparametric statistics. J Am Statistical Ass 1987; 82:645-7.
