# Supplementary material for: Pacemaker Implantation Associated Myocardial Micro-Damage: A Randomised Comparison between Active and Passive Fixation Leads
Source: Sci Rep. 2018 Mar 20;8:4870. doi: 10.1038/s41598-018-23209-5 (PMC5861101; doi:10.1038/s41598-018-23209-5)
Supplement: Supplementary file 3 — Supplementary Tables [file 41598_2018_23209_MOESM3_ESM.pdf]

Pacemaker Implantation Associated Myocardial Micro-Damage:

A Randomised Comparison between Active and Passive Fixation Leads

Dr. med. Patrick Blažek[1], Jerko Ferri-Certić, MD[2], Hrvoje Vražić, MD, PhD[3], Dr. med. Carsten Lennerz[1], Dr. med. univ. Christian Grebmer[1], Kazuaki Kaitani, MD[4], PD Dr. med. Martin Karch[5], Boris Starčević MD, PhD[3], Dr. med. Verena Semmler[1], Prof. Dr. med. Christof Kolb[1]

[1] Deutsches Herzzentrum München, Klinik für Herz- und Kreislauferkrankungen, Abteilung für Elektrophysiologie, Faculty of Medicine Technische Universität München, Munich, Germany
[2] Dubrovnik General Hospital, Dept. of Cardiology, Dubrovnik, Croatia
[3] University Hospital Dubrava, Division of Cardiology, Department of Internal Medicine, Zagreb, Croatia
[4] Tenri Hospital, Dept. of Cardiology, Tenri, Japan
[5] Klinikverbund Kempten-Oberallgäu, Abteilung für Kardiologie, Kempten, Germany
All authors take responsibility for all aspects of the reliability and freedom from bias of the data presented and their discussed interpretation

ClinicalTrials.gov Identifier: NCT01897558
ClinicalTrials.gov Registration date: 12/07/2013

Corresponding author:
Dr. med. Patrick Blažek
Deutsches Herzzentrum München
Lazarettstr. 36
DE - 80636 München
Phone: +49 89 1218 2020
Fax: +49 89 1218 4593
Mail address: blazek@dhm.mhn.de

Supplementary Table S.1. Parameters of ROC analysis for absolute and relative ΔhsTnT as predictor variables for discrimination between active and passive fixation procedure.

	**Absolute Δ hsTnT**	**Relative Δ hsTnT**
**AUC**	0.522	0.558
**95% CI (AUC)**	0.465-0.578	0.498-0.613
p	0.507	0.086
**criterion (cut-off)**	> 0.0061 ng/ml	> 32.7%
**sensitivity**	65.4	73.6
**specificity**	44.5	37.4
**+LR (95% CI)**	1.18 (1.0-1.4)	1.18 (1.0-1.4)
**-LR (95% CI)**	0.78 (0.6-1.0)	0.71 (0.5-1.0)

AUC = Area under the Curve, CI = Confidence Interval, +LR = Positive Likelihood Ratio, -LR= Negative Likelihood Ratio

Supplementary Table S.2. Absolute and relative ΔhsTnT values in groups with active and passive fixation ventricular leads stratified by ventricular lead diameter.

**RVL Diameter**	**Total cohort**		**Active fixation**		**Passive fixation**		***p****
	**N**	**Absolute Δ hsTnT** [ng/ml], *median (IQR)*	**N**	**Absolute Δ hsTnT** [ng/ml], *median (IQR)*	**N**	**Absolute Δ hsTnT** [ng/ml], *median (IQR)*	
< 6 F	65	0.011 (0.005-0.021)	19	0.007 (0.001 – 0.011)	46	0.012 (0.005 – 0.030)	0.030
= 6 F	196	0.077 (0.003-0.018)	111	0.008 (0.004 – 0.020)	85	0.006 (0.002 – 0.016)	0.162
> 6 F	53	0.011 (0.005-0.025)	29	0.015 (0.008 – 0.030)	24	0.008 (0.002 – 0.024)	0.124
p[†]	0.080		0.030		0.070		
	**N**	**Relative Δ hsTnT** [%]	**N**	**Relative Δ hsTnT** [%]	**N**	**Relative Δ hsTnT** [%]	***p****
< 6 F	65	61 (22-152)	19	40 (18- 64)	46	86 (52-138)	0.019
= 6 F	196	55 (19-132)	111	73 (26-171)	85	43 (16-105)	0.010
> 6 F	53	110 (29-267)	29	150 (43-337)	24	50 (16-242)	0.095
p[†]	0.086		0.005		0.036		

RVL = Right-ventricular Lead, hsTnT = High-sensitive Troponin T

Comparisons of values were done between active and passive fixation procedure (Mann-Whitney test, *p**) and within total cohort as well as within each procedure (Kruskal-Wallis test, *p* [†]).

Supplementary Table S.3. Correlation of hsTnT levels with patients' age.

Correlation	**Total cohort**		**Active fixation**		**Passive fixation**	
	r	***p***	***r***	***p***	***r***	***p***
**Baseline hsTnT - Age**	0.437	<0.001	0.435	<0.001	0.445	<0.001
**Post-proc hsTnT - Age**	0.317	<0.001	0.336	<0.001	0.309	<0.001
**Absolute Δ hsTnT - Age**	0.073	0.201	0.107	0.181	0.041	0.609
**Relative Δ hsTnT - Age**	-0.114	0.052	-0.095	0.232	-0.119	0.141

hsTnT = High-sensitive Troponin T

Presented are Spearman rank correlation coefficients *r* and their levels of statistical significance, *p*.
